# Blood Transfusions following Trauma: Finding an Evidence-Based Vein

**DOI:** 10.1371/journal.pmed.1001665

**Published:** 2014-06-17

**Authors:** Druin Burch

**Affiliations:** Public Library of Science, Cambridge, United Kingdom

## Abstract

Druin Burch reflects on new research by Pablo Perel and colleagues and notes that an important fraction of trauma-related deaths could be avoided by improving our knowledge of when to give blood to patients.

*Please see later in the article for the Editors' Summary*

**Linked Research Article**

This Perspective discusses the following new study published in *PLOS Medicine*:

Perel P, Clayton T, Altman DG, Croft P, Douglas I, et al. (2014) Red Blood Cell Transfusion and Mortality in Trauma Patients: Risk-Stratified Analysis of an Observational Study. PLoS Med 11(6): e1001664. doi:10.1371/journal.pmed.1001664

Using a large multicentre cohort, Pablo Perel and colleagues evaluate the association of red blood cell transfusion with mortality according to the predicted risk of death for trauma patients.

Deaths from injuries account for six million deaths a year, a third more than malaria, tuberculosis, and HIV combined. [1] In this week's *PLOS Medicine,* Pablo Perel and colleagues note that haemorrhage kills 30% to 40% of those who die from injuries. Their Research Article suggests that an important fraction of trauma-related deaths could be avoided through simply improving our knowledge of when to give blood to patients suffering trauma [2]. “Although red blood cell (RBC) transfusion is often used in the management of bleeding trauma patients,” they write, “there is considerable uncertainty regarding the balance of risks and benefits.”

Blood transfusion is an expensive intervention that does not always improve clinical outcomes (it can be hard or impossible for an observer to know) and has known hazards, including immunological and infective hazards. Despite being in use for a long time—the rise of blood transfusions for injuries was driven by the First World War, which started a century ago—we know surprisingly little about when best to use them. Indeed, it may be that with transfusion, as with so much else, the phrase “risks and benefits” reinforces the optimistic but misleading medical habit of assuming the existence of benefits and failing to quantify the chance of harms.

The Research Article by Perel and colleagues presents an analysis of data gathered as part of the CRASH-2 trial of tranexamic acid in trauma [3]. CRASH-2 tested an existing intervention for which no company possessed a patent: the mortality reduction the study demonstrated is a good example of the benefits that can be gained from properly testing therapies that have long existed but have never been sufficiently evaluated. RBC transfusion is an obvious next target. It is difficult to justify that medical science oversees the transfusion of 80 million units of blood each year, at a cost per unit of anything from US$40 [4] to US$1,183 [5], all while knowing that doing so can confer serious harms and serious benefits and yet without having reliably investigated how to diminish the former and maximise the latter.

Observational studies have noted that blood transfusion in trauma is associated with poor outcomes, but the potential for confounding is as obvious as the correlation between severe bleeding and severe injury. By using a validated model [6] to categorise patients into four predefined strata of predicted risk of dying (<6%, 6%–20%, 21%–50%, and greater than 50% chance of death) on the basis of initial clinical observations, the current study demonstrates that greater initial likelihood of death is associated with a greater benefit from RBC transfusion. Those at greatest risk of dying experience an odds ratio (OR) of 0.59 (95% confidence interval [CI] 0.47–0.74) for death if they are transfused, while those in the 21%–50% risk group see no significant difference in their OR for dying based on whether they are transfused or not (OR 0.92, 95% CI 0.78–1.08). For those within the lower risk strata, transfusion is associated not with benefit but with harm. Patients at a 6%–20% chance of death had an OR of 2.31 (95% CI 1.96–2.73) for dying if they received a transfusion, while for those whose initial risk was below 6%, the OR for death associated with transfusion was 5.40 (95% CI 4.08–7.13). These are compelling findings.

Our uncertainty about how much blood to give and when to provide it, not only in trauma but also in other settings, has long been known, and recent trials in gastrointestinal haemorrhage [7] orthopaedic surgery [8], and acute myocardial infarction [9] have tended to show that when a liberal and a conservative transfusion strategy are compared across a range of end points, the conservative strategy is better. Using observational data—even with a wealth of care to adjust for known confounders—has limitations. In that sense the study by Perel and colleagues merely reminds us that how much blood to give, and when, is an important area of ignorance. The real originality of the study is in its demonstration that the risk associated with transfusion shifts so considerably when trauma patients are stratified by the severity of their presentation.

One potential mechanism by which transfusion may kill is via vascular occlusive events. Perel and colleagues note that for those at the lowest predicted risk of death, such events had an OR of 4.92 (95% CI 2.80–8.65) associated with RBC transfusion. Other recent work [10] has highlighted that the risk of infection from blood transfusion may not be limited to blood-borne viruses, demonstrating that restrictive transfusion strategies are associated with reduced numbers of subsequent health-care-associated infections. These explorations of why transfusion may cause harm are interesting but do not address what we most need to know: explaining harm is not as important as avoiding it.

The role of haemoglobin and haematocrit measurement in initial management of trauma merits consideration, and was something Perel and coworkers lacked the data to address. As a result, the possibility remains that lower initial measurements of haemoglobin or haematocrit than would be guessed on the basis of the risk strata were a confounder linking the likelihood of blood transfusion and death. Yet soon after trauma (CRASH-2 patients were enrolled within eight hours of injury, the mean being under three hours [3]), haemoglobin and haematocrit measurements are unreliable. Basing transfusion strategies on physiological variables is likely to be, as the authors argue, more useful than basing them on initial laboratory measurements.

RBC transfusions can save lives. “But not,” noted a recent editorial [11] in *JAMA: The Journal of the American Medical Association*, “across the entire range of hemoglobin thresholds currently used to trigger a transfusion.” RBC transfusions can cost lives too, and it is possible, and perhaps likely, that they do so at some of the haemoglobin and clinical thresholds currently used. Trauma and transfusions are too common and too costly for us to tolerate this uncertainty. Although Perel and colleagues describe their article as “informative for current clinical practice,” its value lies in telling us that current clinical practice is dangerously uninformed. It does so in such a way as to persuasively suggest that future trials of transfusion in trauma must take account of the severity of a patient's presentation. Our evidence base for transfusions in trauma is poor. With so many dying each year, and with deaths from injury set to rise in importance as road traffic crashes and violent injuries account for a greater portion of the global burden of disease [12], we have a compelling reason to improve and rationalize our transfusion strategies.

## Abbreviations

CI: confidence interval
OR: odds ratio
RBC: red blood cell
